# Man’s Rights

**Published:** 1873-08

**Authors:** 


### MAN'S RIGHTS.

At any little ant hill there may be seen two lines of the industrious creatures—one going out, the other coming in; and another thing may be seen, very wise in its way: they give way to each other, instinctively, promptly. A coarse man met John Randolph one day; the road was so narrow that one party was compelled to step aside. The countryman promptly took his stand and blurted out, "I never give way to a puppy." "I always do," said Mr. Randolph, and promptly stepped aside.

Suppose any man walks down Broadway with the determination that he will give only half the track to every one he meets. What with the obstruction of some and the hurry of others he will be in a continual jostle, and will be greatly impeded in his way, losing time, temper and politeness. Abstractly he is correct, but the practical working is that, by promptly yielding whenever it is necessary, he will get to his journey's end a great deal sooner, without any rufflement of temper and no bruises next day.

The men who will have their rights, to the very last cent, are theoretically right but practically wrong, and are certainly among the most disagreeable people in the world —ugly in mind, manners and morals, and the less you have to do with them the better. Nobody can possibly respect them, and they are instinctively shunned by all who have ever had anything to do with them.

A day seldom passes wherein circumstances do not occur making it better to "give in"—to yield a little; it promotes neighbourly feeling, and engenders sentiments of cordiality and respect, leaving an impression for generosity and large heartedness which will remain for a lifetime. The moment you see a man willing to give in, that very moment you feel like yielding yourself. But when two unyielding persons meet, the matter of a few cents often creates life-long animosities. Almost every person will find repeated occasions every day in which it is wiser and better to "give in," and not get the unenviable reputation of being "a great stickler for his rights." In fact, it very often happens that men have their eyes so steadily fixed on their own rights, that they cannot see the most palpable rights of others. To be everlastingly contending for our own rights, to be all the time looking out that other people shall not be treading on our own toes, and to be training ourselves up to the necessity of knocking down every man who does, is to cultivate a most unenviable state of mind. A thousand times better is it to take note of the Scripture suggestion, to lead a quiet and peaceable life, and thus have the "wisdom that is peaceable, gentle, and easy to be entreated."

